# Who Are Dentists?

**Published:** 1861-03

**Authors:** Wm. A. Pease


### WHO ARE DENTISTS?

BY WM. A. PEASE.

DENTISTRY as a profession is of American origin. It had its rise in a great public want which nowhere but in the United States has, or could have existed; and there is little likelihood that it ever will exist in any other country. The want that gave it birth, gave it, also, a vigorous growth, unexampled in any other profession, for dentistry can not truly be said to date back much earlier than 1810; and, as a profession, by which is meant the point of time when dentists felt competent to say to the people that, accidents excepted, there is no longer any need for them to lose their teeth, is of as recent date as 1854. As early as 1820 porcelain teeth were made in France, but they were very imperfect imitations of the natural organs; being not very dissimilar in color and general appearance, from common porcelain ware. In this country, Washington bought two sets of artificial teeth, both of which were carved out of solid slabs of ivory.

Up to 1840 a gradual advance in improvement was made, which served as a good foundation for further progress. At this time there was a large class of people in this country, possessed of considerable wealth and occupying high social and official positions, who had lost a part, or the whole of their teeth. This loss was severely felt; not only as a means of masticating their food, as an impediment to articulation, but, also, as destructive of personal beauty and of manly and womanly expression. Hence, the first demand for dentists was to supply the loss of the natural teeth. This want was happily one that appealed to a people generally educated, ingenious and fruitful in devices—to natural mechanics; and it was answered by improvement rapidly following improvement in the art of making artificial sets of teeth, till it was soon brought to very great perfection.

Thus we see that the first want, *for what was, and is still called by many, dental skill*, was for mechanics—persons skilled in working and manipulating gold and silver and fashioning them into plates to fit the mouth; to which teeth were to be attached. This art was justly highly appreciated, because it was very useful, and gave dentists a high position in popular estimation; *it opened the way for dentistry as a profession.*

About 1840 it became obvious that dentistry would soon present a field for honorable and lucrative employment to men of general and medical education. The Baltimore college had just been established, and it was thought that, by scientific investigation, means would soon be found to preserve the natural teeth, and thus greatly diminish, if not entirely dispense with the necessity for artificial substitutes. Then followed an active course of experiments and investigations to determine how far plugs would protect the teeth from further decay. Many of these experiments were necessarily imperfect and ill directed, and it soon became obvious that people had more confidence in mechanical than conservative dentistry, and neglected their teeth. This neglect was ruinous; the nerve became exposed, toothache supervened and forced them to apply to their dentists for relief. The remedy was, therefore, either to extract the tooth, try to quiet and save the nerve, or to destroy it at once. Mechanics chose the former, because it enabled them to make a substitute, dentists the latter, because they considered it an opprobrium to be unable to save the tooth. Their first efforts, it is true, though apparently successful and gratifying, too often ended in a mortifying failure; the capped or treated nerves died, and dead nerves generally produced such a crop of intractable ulcerations, or gumboils, as to disgust the community and dishearten dentists, who then feared that mechanics must still be necessary.

At last, in 1854, a rational theory was promulgated for preventing ulceration and curing ulcerated teeth, and about the same time, as if to fix, and make that the initial point for the advent of dentistry as a profession, several improvements were made in preparing gold for dental purposes that enabled dentists to make much more dense and durable fillings, and also, to build up and restore the form of broken teeth. The dental profession was then fairly established; in theory at least there was no longer any, or but little need of mechanics or manufacturers of artificial sets of teeth; because, that unfortunate class of persons who either ignorantly or criminally neglected their teeth until the nerve became exposed, could still have them treated, plugged, and made much better, and more serviceable than artificial teeth. Now, after a more than six years' practice of the new method of treating diseased teeth, *dentists feel warranted in saying to their patients there is no necessity for losing your teeth.* Teeth can be so thoroughly plugged that it will be as difficult to remove the plug as it would be to cut away a corresponding portion of the tooth. Even in those complicated cases where the tooth aches, where there is a swelling of the face, a gumboil, or a discharge of pus, the pain can still be quieted and the ulcer healed.

From these considerations the public will see there are two classes of dentists, or rather, there are dentists and mechanics, from whom very different treatment may be expected. The one, basing their practice on a knowledge of the human system and the laws that govern it, will preserve the natural teeth; they will refuse to extract them, simply because they ache, or there is a gumboil at the roots; or, if they do it, they will do so reluctantly for persons who persistently demand it, and refuse to submit to the necessary treatment to preserve the teeth; the other, conscious of their inability to treat and save the teeth, having little more than mechanical skill and manual dexterity, will as persistently advise to have the teeth extracted, or they will adroitly lead the patient to demand it by discouraging remarks on the difficulty of the case, or by talking of the danger of ulceration, or what they call disease of the roots; they act on the maxim that dead men and extracted teeth tell no tales. The patients of the first preserve their teeth—their faces unmutilated and their natural expression of countenance and their individuality;—the patrons of the other get a shining set of white teeth, which every one knows to be artificial; in return for which, they are but imperfectly cherished, the breath becomes offensive; the mouth falls in, the nose sticks further out, the lips shorten, the lips and cheeks become wrinkled and shriveled, the cheek bones assume an unnatural prominence, and they look prematurely old. The one, feeling little more responsibility resting upon them than what is common to mechanics, act accordingly; they persistently seek for a sale of their wares, talk loudly and promise much; they obtrusively thrust forward their mechanical contrivances and show pieces of artificial teeth on the attention of the public, all of which the other as studiously avoid. The one never rises above the customs of a craft or trade; the other is governed by the rules of a profession.

